# Supplementary material for: A Likelihood Approach for Real-Time Calibration of Stochastic Compartmental Epidemic Models
Source: PLoS Comput Biol. 2017 Jan 17;13(1):e1005257. doi: 10.1371/journal.pcbi.1005257 (PMC5240920; doi:10.1371/journal.pcbi.1005257)
Supplement: S1 File — (TAR.GZ) [file pcbi.1005257.s014.tar.gz › HSPH_Online-SI-Revision/MSS10/n10-mild/n10-mild_NEW_table4.pdf]

| | | | | | | | | | | | | | | | | | | | | | | | | | | | |
|---|---|---|---|---|---|---|---|---|---|---|---|---|---|---|---|---|---|---|---|---|---|---|---|---|---|---|---|
| 1 | R0 | {1.25046, 6.80278, 0.1} | {1.19168, 2.92811, 0.11} | {{1.06738, 2.19254, 0.}, {1.22544, 3.57392, 1.}} | {4.70779, 56.9572, 188.} | inf | 141. | 59.7982 | {19.9952, 102.909} | 57.5955 | 0.451542 | newly | 135. | 128.58 | {41., 212.} | 38.9778 | 0.219968 | Reff | 0.976984 | 0.940665 | {0.843891, 0.971696} | 3.79448 | ar | 3516. | 2651.1 | {2166., 3148.} | 24.599 |
| 2 | R0 | {1.32013, 4.45847, 0.1} | {1.26849, 3.58683, 0.} | {{1.25474, 3.26445, 0.}, {1.28596, 3.98513, 0.}} | {3.91136, 19.5503, 100.} | inf | 141. | 87.7552 | {52.7683, 131.684} | 38.0201 | 0.239001 | newly | 183. | 138.79 | {79., 199.} | 29.2732 | 0.172012 | Reff | 0.815972 | 0.790785 | {0.78051, 0.802265} | 3.08677 | ar | 4420. | 4067.73 | {3872., 4267.} | 7.99661 |
| 3 | R0 | {1.31785, 5.36796, 1.} | {1.3124, 3.60911, 1.53} | {{1.24751, 2.5364, 0.}, {1.3618, 4.98002, 3.}} | {3.16897, 32.7658, 115.} | inf | 276. | 164.765 | {86.0558, 232.048} | 40.3254 | 0.255736 | newly | 309. | 255.68 | {163., 325.} | 20.3366 | 0.112486 | Reff | 0.782671 | 0.793849 | {0.759493, 0.822273} | 2.97511 | ar | 4942. | 4471.61 | {4127., 4825.} | 9.51821 |
| 4 | R0 | {1.38801, 6.16031, 0.1} | {1.36054, 4.94301, 1.05} | {{1.3618, 4.98002, 1.}, {1.3618, 4.98002, 1.}} | {1.99885, 19.7604, 950.} | inf | 192. | 189.18 | {158.496, 223.519} | 11.2882 | 0.051125 | newly | 196. | 229.31 | {189., 273.} | 18.9745 | 0.0729631 | Reff | 0.811292 | 0.795604 | {0.791241, 0.800427} | 1.93363 | ar | 4790. | 4733.82 | {4595., 4907.} | 2.45887 |
| 5 | R0 | {1.27955, 11.57, 1.} | {1.17082, 5.77111, 1.} | {{1.17082, 5.77111, 1.}, {1.17082, 5.77111, 1.}} | {8.49717, 50.12, 0.} | inf | 157. | 84.0342 | {65.6201, 101.224} | 46.475 | 0.27801 | newly | 94. | 97.93 | {72., 119.} | 16.3511 | 0.0702343 | Reff | 1.02351 | 0.945086 | {0.942973, 0.947132} | 7.66249 | ar | 3460. | 2649.98 | {2384., 2965.} | 23.411 |
| 6 | R0 | {1.22184, 4.34395, 1.} | {1.22805, 3.0708, 0.05} | {{1.22544, 3.07157, 0.}, {1.24751, 3.26445, 0.}} | {0.591964, 29.3086, 95.} | inf | 121. | 70.9817 | {28.9779, 119.815} | 45.3156 | 0.32268 | newly | 169. | 143.26 | {48., 242.} | 38.5562 | 0.229157 | Reff | 0.87777 | 0.888375 | {0.879223, 0.899759} | 1.2831 | ar | 3524. | 3185.28 | {2811., 3542.} | 10.6799 |
| 7 | R0 | {1.23856, 4.59068, 0.1} | {1.24847, 3.10142, 0.03} | {{1.22544, 2.5364, 0.}, {1.25474, 3.26445, 0.}} | {1.28742, 32.441, 128.} | inf | 125. | 85.1642 | {44.5027, 126.12} | 36.3717 | 0.231875 | newly | 186. | 161.2 | {88., 241.} | 29.9355 | 0.169965 | Reff | 0.844822 | 0.856535 | {0.841416, 0.865855} | 1.58077 | ar | 3855. | 3539.74 | {3241., 3840.} | 8.81556 |
| 8 | R0 | {1.34531, 10.6689, 1.} | {1.23782, 3.18039, 0.02} | {{1.22544, 3.07157, 0.}, {1.25474, 3.26445, 0.}} | {7.99016, 70.1901, 98.} | inf | 364. | 101.276 | {54.2905, 145.118} | 72.1769 | 0.592087 | newly | 224. | 194.18 | {110., 282.} | 26.3125 | 0.146298 | Reff | 0.92221 | 0.881043 | {0.867348, 0.898428} | 4.46398 | ar | 4852. | 3425.91 | {3065., 3751.} | 29.3918 |
| 9 | R0 | {1.31775, 5.19226, 0.1} | {1.35972, 4.94834, 1.02} | {{1.3618, 4.98002, 1.}, {1.3618, 4.98002, 1.}} | {3.23409, 4.69783, 922.} | inf | 255. | 222.532 | {178.025, 257.909} | 14.264 | 0.0692531 | newly | 288. | 273.71 | {223., 318.} | 10.941 | 0.0502507 | Reff | 0.778658 | 0.807863 | {0.802775, 0.814462} | 3.77393 | ar | 4708. | 4756.97 | {4578., 4909.} | 2.45603 |
| 10 | R0 | {1.25916, 6.89207, 0.1} | {1.23416, 3.18239, 0.13} | {{1.21159, 3.07157, 0.}, {1.25474, 3.57392, 1.}} | {2.07088, 53.8253, 204.} | inf | 145. | 75.6253 | {44.7781, 111.994} | 47.8446 | 0.306908 | newly | 135. | 142.62 | {89., 211.} | 27.6593 | 0.120269 | Reff | 0.888841 | 0.879748 | {0.865875, 0.895206} | 1.48091 | ar | 3823. | 3293.57 | {3037., 3539.} | 13.8485 |
| 11 | R0 | {1.20377, 5.46566, 0.1} | {1.10709, 2.02091, 2.1} | {{1.06836, 1.78492, 0.}, {1.22544, 3.07157, 4.}} | {8.69169, 63.0254, 2034.} | inf | 117. | 37.8984 | {8.46035, 65.9105} | 67.6082 | 0.60613 | newly | 147. | 96.13 | {20., 178.} | 44.6599 | 0.337548 | Reff | 0.911254 | 0.846783 | {0.81495, 0.934788} | 8.01309 | ar | 3114. | 2554.21 | {2348., 2868.} | 18.3728 |
| 12 | R0 | {1.27878, 2.55804, 0.1} | {1.24751, 2.5364, 0.} | {{1.24751, 2.5364, 0.}, {1.24751, 2.5364, 0.}} | {2.44507, 0.845784, 100.} | inf | 51. | 53.3448 | {25.5068, 81.2537} | 32.8267 | 0.166232 | newly | 100. | 109.68 | {51., 174.} | 37.44 | 0.176829 | Reff | 0.804992 | 0.785017 | {0.78149, 0.788465} | 2.4814 | ar | 3930. | 3885.21 | {3755., 4015.} | 2.58193 |
| 13 | R0 | {1.41227, 5.45696, 0.1} | {1.3618, 4.98002, 1.} | {{1.3618, 4.98002, 1.}, {1.3618, 4.98002, 1.}} | {3.57345, 8.74009, 900.} | inf | 198. | 172.652 | {135.723, 206.976} | 14.8929 | 0.0734105 | newly | 224. | 207.83 | {161., 251.} | 13.1295 | 0.0624699 | Reff | 0.81262 | 0.787033 | {0.782324, 0.791833} | 3.14867 | ar | 4822. | 4750.74 | {4570., 4938.} | 2.79884 |
| 14 | R0 | {1.32917, 7.27593, 1.} | {1.29437, 4.35488, 0.36} | {{1.28596, 3.98513, 0.}, {1.34436, 6.25107, 2.}} | {2.97659, 40.1468, 100.} | inf | 261. | 146.99 | {88.8405, 244.155} | 44.3273 | 0.278829 | newly | 225. | 199.5 | {134., 264.} | 19.6622 | 0.0989684 | Reff | 0.839903 | 0.832573 | {0.824499, 0.849389} | 1.26272 | ar | 4758. | 4127.95 | {3875., 4552.} | 13.4117 |
| 15 | R0 | {1.31629, 9.97481, 2.} | {1.21611, 3.47848, 0.77} | {{1.21159, 3.07157, 0.}, {1.22544, 3.57392, 1.}} | {7.61105, 65.1273, 61.5} | inf | 321. | 101.286 | {56.1497, 145.481} | 68.4468 | 0.539263 | newly | 226. | 187.21 | {105., 259.} | 26.1195 | 0.156073 | Reff | 0.982216 | 0.934183 | {0.925357, 0.945192} | 4.89026 | ar | 4543. | 3027.87 | {2590., 3433.} | 33.3509 |
| 16 | R0 | {1.36735, 5.74166, 0.1} | {1.28538, 3.96319, 0.12} | {{1.28596, 3.98513, 0.}, {1.28596, 3.98513, 0.}} | {5.99489, 30.9748, 208.} | inf | 216. | 141.041 | {107.339, 175.18} | 34.7822 | 0.192919 | newly | 224. | 211.98 | {162., 259.} | 15.4643 | 0.0721653 | Reff | 0.874557 | 0.831761 | {0.826919, 0.837556} | 4.89344 | ar | 4516. | 4057.32 | {3871., 4260.} | 10.1568 |
| 17 | R0 | {1.27524, 13.2396, 0.1} | {1.07341, 2.61007, 0.62} | {{1.06256, 2.19254, 0.}, {1.08063, 3.02112, 1.}} | {15.8271, 80.2858, 606.} | inf | 156. | 37.9191 | {8.34706, 73.8747} | 75.6929 | 0.738575 | newly | 82. | 99.99 | {17., 184.} | 65.5488 | 0.303898 | Reff | 1.15027 | 0.980885 | {0.970694, 0.990132} | 14.7254 | ar | 3731. | 1380.64 | {858., 1973.} | 62.9954 |
| 18 | R0 | {1.21884, 7.71545, 1.} | {1.21188, 3.53045, 0.92} | {{1.21159, 3.57392, 1.}, {1.21159, 3.57392, 1.}} | {0.66876, 54.2418, 10.} | inf | 198. | 87.9776 | {62.2468, 116.254} | 55.5669 | 0.368618 | newly | 194. | 158.94 | {107., 215.} | 23.8763 | 0.127551 | Reff | 0.931559 | 0.939572 | {0.935899, 0.945339} | 1.00301 | ar | 3080. | 2902.38 | {2583., 3223.} | 8.05844 |
| 19 | R0 | {1.34879, 6.71464, 0.1} | {1.25096, 2.58486, 0.2} | {{1.24751, 2.5364, 0.}, {1.24751, 2.5364, 0.}} | {7.25346, 61.5041, 288.} | inf | 164. | 60.5133 | {28.29, 91.3111} | 63.1016 | 0.481067 | newly | 162. | 119.56 | {47., 189.} | 34.0864 | 0.213595 | Reff | 0.798079 | 0.75313 | {0.74754, 0.756521} | 5.63219 | ar | 4585. | 4163.01 | {4017., 4314.} | 9.23991 |
| 20 | R0 | {1.37284, 5.75036, 0.1} | {1.26604, 3.35948, 0.02} | {{1.24751, 2.5364, 0.}, {1.28596, 3.98513, 0.}} | {7.77935, 41.5779, 118.} | inf | 225. | 113.803 | {61.1593, 161.798} | 49.4208 | 0.3195 | newly | 244. | 198.16 | {118., 270.} | 23.9344 | 0.128059 | Reff | 0.87848 | 0.82418 | {0.811505, 0.837456} | 6.18114 | ar | 4750. | 3937.1 | {3703., 4203.} | 17.1137 |
| 21 | R0 | {1.29938, 5.36419, 0.1} | {1.26952, 3.57703, 0.04} | {{1.25474, 3.26445, 0.}, {1.28596, 3.98513, 0.}} | {2.32598, 33.3165, 136.} | inf | 178. | 106.586 | {64.5088, 137.369} | 40.2517 | 0.240824 | newly | 209. | 171.56 | {108., 226.} | 22.6411 | 0.119661 | Reff | 0.808604 | 0.799064 | {0.787529, 0.8102} | 1.34908 | ar | 4337. | 4068.12 | {3875., 4239.} | 6.2006 |
| 22 | R0 | {1.31882, 7.47626, 0.1} | {1.22431, 3.25321, 0.33} | {{1.21159, 3.07157, 0.}, {1.25474, 3.57392, 1.}} | {7.16604, 56.4861, 364.} | inf | 191. | 76.8287 | {33.0319, 120.421} | 59.7756 | 0.439434 | newly | 169. | 143.54 | {58., 224.} | 30.8402 | 0.172871 | Reff | 0.929504 | 0.876875 | {0.86517, 0.893196} | 5.66206 | ar | 3977. | 3234.13 | {2920., 3515.} | 18.7013 |
| 23 | R0 | {1.26215, 3.46375, 0.1} | {1.2421, 3.00346, 0.01} | {{1.22544, 2.5364, 0.}, {1.25474, 3.26445, 0.}} | {1.58849, 13.3523, 108.} | inf | 114. | 75.672 | {31.7759, 115.061} | 35.9543 | 0.233673 | newly | 207. | 146.62 | {62., 228.} | 34.2415 | 0.221289 | Reff | 0.848291 | 0.83958 | {0.825577, 0.850835} | 1.16788 | ar | 3607. | 3568.83 | {3319., 3816.} | 4.25645 |
| 24 | R0 | {1.16712, 4.19464, 1.} | {1.22584, 2.36274, 1.04} | {{1.19264, 2.01465, 0.}, {1.24751, 2.5364, 3.}} | {5.35565, 43.6723, 134.} | inf | 82. | 44.1517 | {16.5045, 80.4669} | 48.4053 | 0.358093 | newly | 105. | 113.36 | {40., 208.} | 47.9429 | 0.22481 | Reff | 0.873473 | 0.922048 | {0.895363, 0.94117} | 5.85344 | ar | 3000. | 2843. | {2505., 3226.} | 8.702 |
| 25 | R0 | {1.22751, 3.34517, 0.1} | {1.23654, 2.43205, 0.6} | {{1.19264, 2.01465, 0.}, {1.24751, 2.5364, 3.}} | {1.8718, 27.2966, 660.} | inf | 114. | 117.873 | {54.7461, 178.361} | 32.7989 | 0.157836 | newly | 241. | 300.98 | {156., 443.} | 43.0456 | 0.170706 | Reff | 0.932662 | 0.939025 | {0.907072, 0.952849} | 1.75276 | ar | 3086. | 3270.37 | {2866., 3695.} | 9.83377 |
| 26 | R0 | {1.18747, 5.88083, 1.} | {1.24611, 2.58843, 0.} | {{1.24751, 2.5364, 0.}, {1.24751, 2.5364, 0.}} | {4.93841, 55.9853, 100.} | inf | 107. | 48.6639 | {13.212, 81.1383} | 54.5858 | 0.426899 | newly | 102. | 105.17 | {31., 175.} | 44.4804 | 0.235436 | Reff | 0.802017 | 0.848894 | {0.842805, 0.853709} | 5.84491 | ar | 3614. | 3419.49 | {3187., 3636.} | 6.02241 |
| 27 | R0 | {1.29023, 4.76202, 0.1} | {1.2966, 4.09461, 0.86} | {{1.28596, 3.98513, 0.}, {1.31748, 4.25564, 2.}} | {0.871045, 14.0154, 874.} | inf | 148. | 124.066 | {95.6939, 151.424} | 18.8095 | 0.0950594 | newly | 192. | 177.57 | {135., 219.} | 16.0156 | 0.0764569 | Reff | 0.787814 | 0.794802 | {0.785792, 0.80821} | 1.02007 | ar | 4245. | 4282.71 | {4098., 4422.} | 2.21178 |
| 28 | R0 | {1.2258, 12.9056, 3.} | {1.20299, 3.73531, 1.09} | {{1.17082, 3.57392, 1.}, {1.21159, 3.74835, 1.}} | {1.8607, 71.0567, 65.} | inf | 171. | 51.1477 | {22.3439, 76.1301} | 70.0891 | 0.576926 | newly | 91. | 89.61 | {38., 138.} | 35.2418 | 0.183551 | Reff | 0.929156 | 0.926289 | {0.898593, 0.936399} | 1.05764 | ar | 3639. | 2700.34 | {2380., 3014.} | 25.7944 |
| 29 | R0 | {1.34702, 6.29049, 0.1} | {1.24964, 2.6015, 0.08} | {{1.24751, 2.5364, 0.}, {1.24751, 2.5364, 0.}} | {7.22911, 58.6439, 174.} | inf | 220. | 111.26 | {64.4586, 153.435} | 49.4273 | 0.329099 | newly | 234. | 224.71 | {133., 318.} | 23.4573 | 0.120267 | Reff | 0.838924 | 0.791856 | {0.785625, 0.796878} | 5.61051 | ar | 4560. | 4033.5 | {3841., 4265.} | 11.5461 |
| 30 | R0 | {1.37931, 6.78741, 1.} | {1.29192, 3.62367, 1.1} | {{1.24751, 2.5364, 0.}, {1.31748, 4.25564, 3.}} | {6.33588, 46.6119, 114.} | inf | 216. | 110.483 | {51.5337, 154.847} | 48.8506 | 0.326028 | newly | 206. | 169.26 | {105., 223.} | 23.2816 | 0.12968 | Reff | 0.813793 | 0.775809 | {0.75275, 0.79075} | 4.72252 | ar | 4667. | 4330.53 | {4131., 4557.} | 7.22413 |
| 31 | R0 | {1.16062, 4.47529, 0.1} | {1.23133, 2.96657, 0.01} | {{1.22544, 2.5364, 0.}, {1.24751, 3.07157, 0.}} | {6.0922, 33.7121, 108.} | inf | 144. | 76.9711 | {33.8433, 114.357} | 47.6044 | 0.328056 | newly | 207. | 167.35 | {90., 263.} | 31.9179 | 0.191605 | Reff | 0.868956 | 0.930152 | {0.921117, 0.945142} | 7.04246 | ar | 3037. | 3057.36 | {2761., 3325.} | 6.3622 |
| 32 | R0 | {1.37647, 18.7122, 2.} | {1.21798, 4.01511, 0.99} | {{1.21159, 3.07157, 0.}, {1.22544, 8.76728, 4.}} | {11.5142, 78.5428, 74.5} | inf | 353. | 67.1031 | {28.6135, 149.315} | 80.9906 | 0.8017 | newly | 116. | 100.99 | {51., 142.} | 27.9569 | 0.157704 | Reff | 0.957197 | 0.881807 | {0.870591, 0.891828} | 7.87612 | ar | 4824. | 3136.58 | {2865., 3522.} | 34.9797 |
| 33 | R0 | {1.35084, 6.08841, 0.1} | {1.36136, 4.97277, 1.01} | {{1.3618, 4.98002, 1.}, {1.3618, 4.98002, 1.}} | {0.828167, 18.3239, 910.} | inf | 251. | 191.604 | {161.344, 222.324} | 23.6636 | 0.120846 | newly | 266. | 231.18 | {190., 278.} | 15.2331 | 0.0741997 | Reff | 0.778759 | 0.792909 | {0.788634, 0.797188} | 1.83651 | ar | 4759. | 4746.69 | {4557., 4946.} | 2.39399 |
| 34 | R0 | {1.36912, 6.316, 1.} | {1.24729, 2.54176, 0.} | {{1.24751, 2.5364, 0.}, {1.24751, 2.5364, 0.}} | {8.89825, 59.7569, 100.} | inf | 243. | 85.0711 | {35.4371, 133.014} | 64.9913 | 0.515361 | newly | 247. | 192.14 | {74., 302.} | 32.6235 | 0.206835 | Reff | 0.921965 | 0.859625 | {0.853718, 0.865936} | 6.76171 | ar | 4537. | 3529.19 | {3200., 3844.} | 22.2131 |
| 35 | R0 | {1.35831, 7.09038, 1.} | {1.2926, 4.05574, 0.56} | {{1.28596, 3.98513, 0.}, {1.31748, 4.25564, 2.}} | {4.83765, 42.7994, 100.} | inf | 210. | 106.654 | {78.3133, 133.897} | 49.2122 | 0.305708 | newly | 184. | 152.55 | {111., 196.} | 20.7446 | 0.108836 | Reff | 0.809553 | 0.783744 | {0.776779, 0.796029} | 3.18802 | ar | 4667. | 4271.34 | {4096., 4444.} | 8.48982 |
| 36 | R0 | {1.28315, 5.89938, 0.1} | {1.24751, 2.5364, 0.} | {{1.24751, 2.5364, 0.}, {1.24751, 2.5364, 0.}} | {2.77731, 57.0056, 100.} | inf | 195. | 77.1379 | {36.3862, 112.368} | 60.4421 | 0.437919 | newly | 206. | 156.36 | {73., 231.} | 31.301 | 0.184592 | Reff | 0.765399 | 0.758845 | {0.754441, 0.763813} | 0.85783 | ar | 4460. | 4145.09 | {3980., 4322.} | 7.06794 |
| 37 | R0 | {1.19303, 2.75113, 0.1} | {1.20315, 2.13323, 2.29} | {{1.19264, 2.01465, 0.}, {1.24751, 2.5364, 3.}} | {1.34398, 22.4598, 2238.} | inf | 59. | 45.3893 | {12.3703, 85.2576} | 42.9314 | 0.295693 | newly | 124. | 118.54 | {31., 214.} | 47.2742 | 0.288141 | Reff | 0.868407 | 0.8774 | {0.865058, 0.910212} | 1.58386 | ar | 3142. | 2972.83 | {2702., 3267.} | 7.55029 |
| 38 | R0 | {1.36033, 7.8088, 1.} | {1.2676, 3.25869, 0.36} | {{1.25474, 1.35944, 0.}, {1.28596, 3.98513, 3.}} | {6.81671, 58.269, 112.} | inf | 289. | 121.895 | {70.8328, 164.362} | 57.8218 | 0.396952 | newly | 222. | 220.26 | {122., 282.} | 23.982 | 0.109895 | Reff | 0.859593 | 0.822173 | {0.80976, 0.8349} | 4.35314 | ar | 4941. | 3980.85 | {3722., 4222.} | 19.4323 |
| 39 | R0 | {1.27353, 3.70845, 0.1} | {1.24808, 2.64903, 0.} | {{1.24751, 2.5364, 0.}, {1.25474, 3.26445, 0.}} | {1.99814, 28.5676, 100.} | inf | 54. | 54.1322 | {20.5652, 82.2854} | 34.0773 | 0.172124 | newly | 83. | 110.29 | {43., 171.} | 55.2169 | 0.209269 | Reff | 0.815441 | 0.79913 | {0.795145, 0.803} | 2.00031 | ar | 3725. | 3782.59 | {3632., 3954.} | 2.80403 |
| 40 | R0 | {1.19983, 12.1486, 0.1} | {1.17082, 5.77111, 1.} | {{1.17082, 5.77111, 1.}, {1.17082, 5.77111, 1.}} | {2.41747, 52.4957, 900.} | inf | 143. | 62.0488 | {44.8572, 78.7628} | 56.6093 | 0.373277 | newly | 81. | 71.11 | {50., 93.} | 20.1358 | 0.101096 | Reff | 0.938027 | 0.924829 | {0.922821, 0.926811} | 1.40705 | ar | 3619. | 2598.53 | {2334., 2900.} | 28.1976 |
| 41 | R0 | {1.20935, 2.67169, 0.1} | {1.24964, 2.35394, 0.52} | {{1.24751, 1.35135, 0.}, {1.2663, 2.5364, 3.}} | {3.38692, 11.8934, 586.} | inf | 33. | 39.4895 | {6.58706, 76.6694} | 69.5134 | 0.333655 | newly | 73. | 94.08 | {15., 186.} | 79.5068 | 0.373562 | Reff | 0.803008 | 0.828949 | {0.822545, 0.838081} | 3.28649 | ar | 3429. | 3532.6 | {3345., 3770.} | 4.30038 |
| 42 | R0 | {1.23313, 4.74781, 1.} | {1.26244, 3.565, 0.08} | {{1.22544, 3.07157, 0.}, {1.28596, 3.98513, 0.}} | {2.70093, 24.9127, 96.} | inf | 154. | 103.289 | {64.7902, 143.702} | 33.7619 | 0.203415 | newly | 207. | 177.18 | {105., 233.} | 23.401 | 0.127643 | Reff | 0.853203 | 0.879845 | {0.857027, 0.897135} | 3.22417 | ar | 3835. | 3555.88 | {3233., 3870.} | 8.06206 |
| 43 | R0 | {1.26105, 2.39713, 0.1} | {1.24938, 2.51197, 0.07} | {{1.24751, 2.5364, 0.}, {1.24751, 2.5364, 0.}} | {1.15703, 6.48075, 166.} | inf | 51. | 91.1991 | {50.5629, 127.174} | 83.067 | 0.250082 | newly | 120. | 193.39 | {103., 288.} | 68.025 | 0.216501 | Reff | 0.808711 | 0.796205 | {0.790351, 0.800206} | 1.75538 | ar | 3723. | 3938.97 | {3725., 4142.} | 6.02981 |
| 44 | R0 | {1.36948, 8.88006, 0.1} | {1.23706, 3.24504, 0.12} | {{1.21159, 3.07157, 0.}, {1.25474, 3.57392, 1.}} | {9.66953, 63.457, 196.} | inf | 313. | 104.427 | {65.4079, 141.594} | 66.6369 | 0.496097 | newly | 232. | 195.2 | {130., 266.} | 23.0776 | 0.123808 | Reff | 0.929192 | 0.865139 | {0.849169, 0.881005} | 6.89338 | ar | 4437. | 3521.75 | {3250., 3767.} | 20.6277 |
| 45 | R0 | {1.34882, 3.3791, 0.1} | {1.26227, 2.63644, 0.54} | {{1.24751, 2.5364, 0.}, {1.30094, 3.11158, 3.}} | {6.60257, 21.9781, 604.} | inf | 128. | 93.8177 | {44.1893, 145.838} | 33.6729 | 0.202808 | newly | 208. | 180.99 | {82., 269.} | 29.7837 | 0.163305 | Reff | 0.796074 | 0.74929 | {0.734862, 0.772544} | 6.11919 | ar | 4422. | 4323.55 | {4145., 4517.} | 3.35165 |
| 46 | R0 | {1.23774, 4.34917, 0.1} | {1.26974, 3.61085, 0.} | {{1.24751, 3.07157, 0.}, {1.28596, 3.98513, 0.}} | {2.72431, 16.9762, 100.} | inf | 149. | 136.255 | {92.5671, 172.968} | 18.5977 | 0.0907093 | newly | 217. | 231.18 | {162., 290.} | 20.1106 | 0.0854213 | Reff | 0.844634 | 0.868055 | {0.852805, 0.880787} | 2.81186 | ar | 3685. | 3818.74 | {3552., 4132.} | 5.61465 |
| 47 | R0 | {1.34327, 4.78811, 0.1} | {1.25351, 3.06766, 0.} | {{1.24751, 2.5364, 0.}, {1.25474, 3.26445, 0.}} | {6.68236, 35.9318, 100.} | inf | 220. | 149.482 | {91.086, 205.513} | 32.5439 | 0.18899 | newly | 304. | 277.82 | {182., 371.} | 19.8421 | 0.0974132 | Reff | 0.872454 | 0.822981 | {0.816105, 0.828665} | 5.67055 | ar | 4517. | 3968.28 | {3690., 4223.} | 12.1479 |
| 48 | R0 | {1.24366, 4.25814, 0.1} | {1.24925, 2.35579, 0.54} | {{1.24751, 1.35135, 0.}, {1.2663, 2.5364, 3.}} | {0.725262, 44.6757, 604.} | inf | 141. | 70.3007 | {30.7867, 106.461} | 50.7628 | 0.360231 | newly | 200. | 173.2 | {75., 265.} | 32.53 | 0.180128 | Reff | 0.862976 | 0.875698 | {0.868523, 0.888992} | 1.67248 | ar | 3678. | 3385.16 | {3112., 3652.} | 8.56117 |
| 49 | R0 | {1.36017, 7.5415, 1.} | {1.22509, 3.16756, 0.2} | {{1.21159, 3.07157, 0.}, {1.22544, 3.57392, 1.}} | {9.9312, 57.9983, 80.} | inf | 178. | 83.3705 | {42.4492, 127.196} | 53.1626 | 0.361789 | newly | 164. | 167.99 | {90., 254.} | 30.4207 | 0.137851 | Reff | 1.01999 | 0.930291 | {0.920037, 0.937515} | 8.79426 | ar | 4178. | 3085.74 | {2658., 3432.} | 26.1431 |
| 50 | R0 | {1.25333, 6.63578, 1.} | {1.22803, 3.05882, 0.03} | {{1.22544, 3.07157, 0.}, {1.24751, 3.07157, 0.}} | {2.0297, 53.9041, 97.} | inf | 109. | 46.953 | {14.2025, 72.7335} | 56.9238 | 0.436079 | newly | 93. | 92.24 | {32., 139.} | 37.7634 | 0.201709 | Reff | 0.878334 | 0.868226 | {0.862502, 0.880021} | 1.31651 | ar | 3401. | 3174.29 | {2945., 3401.} | 7.17818 |
